# Burnout and well-being of healthcare workers in the post-pandemic period of COVID-19: a perspective from the job demands-resources model

**DOI:** 10.1186/s12913-022-07608-z

**Published:** 2022-03-02

**Authors:** Ting Zhou, Changshun Xu, Cunliang Wang, Sha Sha, Zhe Wang, You Zhou, Xinran Zhang, Die Hu, Yinqi Liu, Tengfei Tian, Sixiang Liang, Li Zhou, Qian Wang

**Affiliations:** 1 Department of Medical Psychology, School of Health Humanities, Peking University, No.38 Xueyuan Road, Haidian District, Beijing, 100191 China; 2 Beijing Hospitals Authority, No.70 Zaolinqian Road, Xicheng District, Beijing, 100053 China; 3 Beijing Anding Hospital of Capital Medical University, No.5 Ankang Hutong, Deshengmenwai Street, Xicheng District, Beijing, 100088 China; 4 New York Psychoanalytic Society & Insititute, New York, USA

**Keywords:** Healthcare workers, Well-being, Epidemic-related job stressors, Social support, Organizational support

## Abstract

**Background:**

The present study aimed 1) to examine the effects of epidemic-related job stressors, perceived social support and organizational support on the burnout and well-being of Chinese healthcare workers in the period of COVID-19 regular epidemic prevention and control and 2) to investigate the moderating effects of social support and organizational support on the relationship between job stressors and burnout and well-being within the theoretical framework of the Job Demands-Resources (JD-R) model.

**Methods:**

A sample of healthcare workers (*N* = 3477) from 22 hospitals in Beijing, China participated in the cross-sectional investigation in October 2020 and reported epidemic-related job stressors, perceived social support, organizational support, burnout, anxiety and depression symptoms.

**Results:**

1) Medical doctors, females, people aged from 30 to 50, and those who worked in the second line during the pandemic reported higher scores of psychological symptoms and burnout in the period of regular epidemic prevention and control; 2) Epidemic-related job stressors positively predicted burnout, anxiety, and depression among healthcare workers; 3) Perceived social support and organizational support were negatively related to reported burnout, anxiety and depression symptoms; 4) Social support reduced the adverse effects of epidemic-related job stressors on anxiety and depression but enhanced the association between stressors and burnout; 5) Organizational support mitigated the adverse effects of epidemic-related job stressors on depression.

**Conclusion:**

The results shed light on preventing burnout and enhancing the psychological well-being of healthcare workers under epidemic prevention and control measures by reducing epidemic-related job stressors and strengthening personal and organizational support systems.

## Introduction

The outbreak of coronavirus disease 2019 (COVID-19) has resulted in great demand for public health resources and infection control measures worldwide [1]. As the first country in which the COVID-19 outbreak occurred, China had effectively brought the epidemic under control by June 2020 and entered the post-pandemic period. Given the severity of the epidemic abroad, the Chinese government coordinated epidemic prevention and control with the recovery of the economy and production and declared a new stage of regular epidemic prevention and control.

Healthcare workers continue taking on important responsibilities in the new stage of regular epidemic prevention and control. First, infection prevention and control measures in hospitals are vital to prevent the spread of the coronavirus. Second, healthcare workers are responsible for screening patients in their daily work. Third, they need to be well prepared to treat patients with COVID-19 if there are new cases. Given that the pandemic has lasted a year and that prevention and control measures might last longer, it is important to investigate occupational stress and psychological well-being in healthcare workers under the impact of COVID-19 regular epidemic prevention and control measures as well as to identify effective resource management to mitigate the adverse effect of epidemic-related job stressors.

### Job-related stress and psychological impact of COVID-19 on healthcare workers

A large number of studies have been carried out on the occupational stress and well-being of healthcare workers in the era of COVID-19. As found by most studies, healthcare workers experienced heightened levels of psychological symptoms, including anxiety, depression, somatic symptoms, and burnout, during the outbreak of COVID-19 [2–11]. High workload, perceived severity of COVID-19, predictable shortages of supplies, being unable to provide competent medical care, concerns about being infected, and worries about the health and safety of family members and the patients all contribute to pressures on healthcare workers [6–16].

However, most studies have been conducted during the outbreak stage whereas research in the post-pandemic period on job stress and psychological adaptation in healthcare workers is scarce. Informed by research findings of previous major epidemics, work experience in epidemics could have long-term effects on the psychological well-being of healthcare workers [17]. Considering the severity and the protracted timeline, the post-pandemic effect of COVID-19 might exceed those observed in previous pandemics. Thus, it is necessary to examine the long-term effect of work experience during the outbreak period of COVID-19 on the psychological well-being of healthcare workers after the pandemic.

In addition, it is important to note that the job stressors of Chinese healthcare workers in the current regular epidemic control and prevention stage could be very different. Given the dramatic decrease in confirmed cases, the risk of infection has been reduced, and the situation of a shortage of personal protection measures has been improved. However, there is still great uncertainty regarding infection in the workplace of healthcare workers. Fear of infection, which was one of the main stressors for healthcare workers during the out-break period, might not disappear quickly and continue to affect the psychological health of some individuals [18]. Regular epidemic prevention and control measures might also continue placing a burden on healthcare workers [19]. The present study aimed to examine the effects of job stressors of Chinese healthcare workers in regular epidemic prevention and control periods on their burnout and well-being. In addition to job stressors, we also explored how to provide resources to protect the mental health of healthcare workers. The job demands-resources (JD-R) model was used to examine the research questions.

### Job demands-resources model (JD-R)

The JD-R model suggests that working conditions or all aspects in the working environments can be classified as job demands and job resources. For individuals, a strain is a response to an imbalance between job demands and job resources. There has been empirical evidence that job demands and resources are both important predictors of burnout [20–22].

### Job demands

Job demands usually refer to aspects of the job that require sustained physical or psychological effort or skills and are therefore associated with certain physiological or psychological costs, such as high work pressure, job insecurity, and conflict with others [23, 24]. High job demands lead to burnout, which refers to a state of exhaustion and cynicism toward work. It subsequently contributes to negative psychological health [25].

In the context of COVID-19 infection prevention and control measures, healthcare workers might face some specific new demands. For example, healthcare workers have to integrate epidemic investigation, virus testing, and self-protection measurements into their everyday work. Some of them had reported that they were already tired of using protective measures [26]. There is also evidence suggesting that infection prevention and control measures reduce the autonomy of healthcare workers and contribute to their burnout [27]. Healthcare workers need to prepare for changes in work routines due to changes in epidemic trends. In addition, although there are few confirmed cases, healthcare workers might still be affected by worries about infection [19]. Furthermore, there could be interpersonal stressors when communicating infection prevention and control measures with colleagues, supervisors, and patients [28]. It is, therefore, reasonable to hypothesize that new job stressors might impact the psychological well-being of healthcare workers in the new period.

### Job resources

Job resources are defined as aspects of the job that may do any of the following: “(a) be functional in achieving work goals; (b) reduce job demands and the associated physiological and psychological costs; (c) stimulate personal growth and development”. It can be located at the level of the organization at large, interpersonal and social relations, the organization of work, and at the level of the task [23]. Job resources have motivating effects, contribute to high work engagement [25], and buffer the negative effects of excessive job demands on employee health and well-being [22, 29].

Social support has been considered an important resource to cope with job-related stressors [30]. Previous research showed that social support is positively related to well-being but negatively related to burnout among healthcare workers during the COVID-19 pandemic [31], and perceived lack of social support was directly associated with depression, anxiety, stress, and inadequate sleeping [32]. The moderating effects of social support on job-related stress have also been proven in other populations, although not under the situation of COVID-19 [33]. Therefore, social support was hypothesized to be negatively associated with burnout, anxiety, and depression while moderating the effect of epidemic-related job stressors and burnout and well-being for healthcare workers during the COVID-19 pandemic.

As argued by many researchers, organizational support might have particularly important protective effects for healthcare workers in circumstances of epidemics [34–38]. There could be several reasons. First, only organizations are capable of providing adequate effective personal protection equipment and training on the prevention and treatment of diseases. Second, healthcare workers could be encouraged or supported in a supportive organizational climate. A handful of empirical studies examined the effect of perceived organizational support of healthcare workers in the outbreak of COVID-19, and the results supported the beneficial effects of perceived organizational support on job satisfaction [38] and lower levels of anxiety [39]. Although there is no empirical evidence of the moderating effect of organizational support during COVID-19, meta-analysis research of studies over 20 years concluded that organizational support is an important moderator of work performance [40]. Thus, it is reasonable to hypothesize that perceived organizational support would negatively predict burnout and psychological symptoms and buffer the effects of epidemic-related job stressors on the psychological well-being of healthcare workers.

### Overview of the present study

**Fig. 1:**
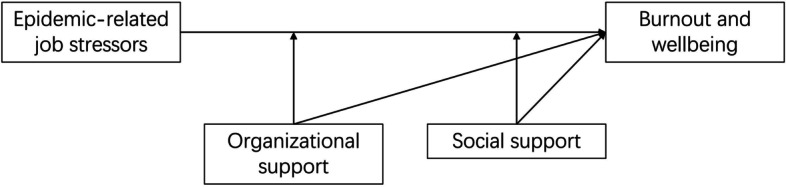
The hypothesized model on associations among epidemic job stressors, organizational support, social support and wellbeing for healthcare workers

Based on the JD-R model, the present study aimed to examine the effects of epidemic-related job stressors (job demands) and perceived social support and organizational support (job resources) on the burnout and well-being of Chinese healthcare workers in the post-pandemic period of COVID-19. According to the theoretical model and results of previous empirical studies, the hypotheses were proposed as follows: 1) epidemic-related job stressors would positively predict burnout, anxiety, and depression symptoms; 2) perceived social support and organizational support would have positive effects on burnout, anxiety, and depression; and 3) perceived social support and organizational support would mitigate the adverse effects of epidemic-related job stressors on burnout, anxiety, and depression. The hypothesized model was illustrated in Fig. 1.

## Materials and methods

### Participants

A total of 3477 healthcare workers from 22 tertiary hospitals in Beijing, China participated in this study. Among them, 829 (23.8%) were doctors, 1794 (51.6%) were nurses, 580 (16.7%) were medical technicians and 274 (7.9%) were administrators. The total sample comprised 760 males (21.9%) and 2717 females (78.1%). The age of the participants ranged from 21 to 61 years old, with an average age of 36.59 years old (SD = 8.17). There were 820 (23.58%) participants working in departments with a higher exposure risk of the coronavirus, such as the infectious disease department, respiratory department, fever clinic, emergency department, intensive care unit, and COVID-19 wards in their hospitals (identified as the higher exposure risk group). One thousand eight hundred ninety (54.36%) participants worked in other departments of the hospital and had no experience taking part in medical rescue teams for combating COVID-19 (identified as the lower exposure risk group). Seven hundred (20.13%) participants took part in medical rescue work in June 2020 when a small epidemic in Beijing emerged (identified as medical team members in a small epidemic in Beijing). There were also 67 (1.93%) participants who took part in the medical teams during the outbreak period in Hubei from January 2020 to May 2020 (identified as medical team members in the Hubei outbreak). The specific demographic characteristics of each group of participants are shown in Table 1.

**Table 1:** Demographic characteristics of participants

	Lower exposure risk group (*n* = 1890)	Higher exposure risk group (*n* = 820)	Medical team members in a small epidemic in Beijing (*n* = 700)	Medical team members in the Hubei outbreak (*n* = 67)
Gender				
Male	459 (24.3%)	135 (16.5%)	143 (20.4%)	23 (34.3%)
Female	1431 (75.7%)	685 (83.5%)	557 (79.6%)	44 (65.7%)
Age				
< 30 y	308 (16.3%)	200 (24.4%)	188 (26.9%)	8 (11.9%)
30–40 y	869 (46.0%)	444 (54.1%)	402 (57.4%)	39 (58.2)
41–50 y	500 (26.5%)	158 (19.3%)	103 (14.7%)	17 (25.4%)
> 50 y	213 (11.2%)	18 (2.2%)	7 (1%)	3 (4.5%)
M	38.33	34.75	33.92	37.52
SD	8.70	7.36	6.27	7.68
Professions				
Doctors	480 (25.4%)	252 (31.1%)	80 (11.4%)	17 (25.4%)
Nurses	676 (35.8%)	503 (62.1%)	567 (81%)	48 (71.6%)
Medical technicians	483 (25.7%)	51 (6.3%)	45 (6.4%)	1 (1.5%)
Administrative staff	251 (13.3%)	4 (0.5%)	8 (1.1%)	1 (1.5%)
Length of working experience				
< 5 y	239 (12.9%)	148 (18.2%)	81 (11.6%)	3 (4.5%)
5–10 y	440 (23.8%)	248 (30.5%)	270 (38.8%)	18 (26.9%)
11–20 y	603 (32.7%)	286 (35.1%)	264 (37.9%)	31 (46.3%)
21–30 y	386 (20.9%)	108 (13.3%)	77 (11.1%)	12 (17.9%)
> 30 y	178 (9.6%)	24 (2.9%)	4 (0.6%)	3 (4.5%)
M	15.86	12.26	11.92	15.37
SD	9.93	7.95	6.70	8.24
Marriage				
Unmarried	276 (14.6%)	242 (29.5%)	241 (34.0%)	15 (22.4%)
Married	1545 (81.7%)	551 (67.2%)	439 (61.9%)	50 (74.6%)
Divorced	66 (3.5%)	26 (3.2%)	27 (3.8%)	2 (3.0%)
Other	3 (0.2%)	1 (0.1%)	2 (0.3%)	0 (0%)
Annual income (CNY)				
< 200 k	1223 (64.7%)	561 (68.4%)	449 (64.1%)	41 (61.2%)
200–300 k	602 (31.9%)	244 (29.8%)	241 (34.4%)	22 (32.8%)
300–500 k	57 (3%)	13 (1.6%)	9 (1.3%)	3 (4.5%)
>500 k	8 (0.4%)	2 (0.2%)	1 (0.1%)	1 (1.5%)

Note. Average annual income of employed persons in urban area of China in 2019 = 90.5 k CNY. (http://data.stats.gov.cn)

### Measures

Basic demographic information of participants, such as gender, age, career, specialty, length of working experience, income and working arrangements during the COVID-19 pandemic, was included in the questionnaire. Job stressors under regular epidemic prevention and control were measured using the newly developed Epidemic-Related Job Stressors Scale. Social support was measured by the Multidimensional Scale of Perceived Social Support, and organizational support was measured by the self-developed Perceived Organizational Support Scale. The well-being of the healthcare workers was measured by the widely used Patient Health Questionnaire (PHQ-9) and Generalized Anxiety Disorder Assessment (GAD-7). Burnout was measured using the Maslach Burnout Inventory-General Scale (MBI-GS).

#### The epidemic-related job stressors scale

Based on interview data collected from healthcare workers, this scale was specifically developed to measure the job stressors of healthcare workers in the period of COVID-19 epidemic regular prevention and control [28]. The 14-item scale consists of three dimensions: stressors related to infection prevention and control measures (9 items), high workload (3 items), and infection anxiety (2 items). The subscales of stressors under infection prevention and control measures include discomfort caused by personal protection equipment (e.g., “The level of personal protection in daily work is too high, which causes unnecessary trouble”; “The level of personal protection in daily work is not adequate to prevent the spread of the virus”), interpersonal stressors when communicating infection and control measures with col-leagues, supervisors, and patients (e.g., “The division of responsibilities between doctors and nurses is ambiguous”; “I am worried about being blamed by the supervisor”), and perceived incompetence on carrying out infection prevention and control measures (e.g., “I am stressed because I am not competent to carry on the infection prevention and control measures”). The subscale of high workload measures stressors concerns with increased work intensity (e.g., “The work routine in this period becomes more complicated. The epidemic history screen increases the workload”). The subscale of infection anxiety comprises two items to measure worries about being infected (e.g., “When I have a fever or cough, I am worried about being infected by the novel coronavirus”). Participants were asked to rate all the items on a 7-point Likert scale (1 = very strongly disagree, 7 = very strongly agree). The total score was used in the data analyses, with a higher score indicating a higher level of job stressors. The reliability and validity of this scale were acceptable [28]. The Cronbach’s α of the whole scale and the three subscales ranged from 0.74 to 0.89 in the present study.

#### The multidimensional scale of perceived social support

The scale contains 12 items with three subscales addressing perceived social support from family, friends, and other important others [41]. Participants were asked to rate those items on a 7-point Likert scale (1 = very strongly disagree, 7 = very strongly agree). The total score was used in the data analyses, with a higher score indicating a higher level of social support. The Chinese version of this scale has good reliability and validity, and it has been widely used in previous research [42]. The Cronbach’s α of the scale in the present study was 0.96.

#### The perceived organizational support scale

The measure was developed based on the existing measurements of organizational support [43, 44] and a description of supportive measures provided by hospitals in previous pandemics [37]. There are 9 items to measure organizational support perceived by healthcare workers from three aspects, namely, instrumental support (“The hospital has provided adequate protective materials and equipment.”), emotional support (“My contribution in fighting against the epidemics was recognized and valued by my organization.”) and institutional protection (e.g., “The hospital provided clear instructions on the diagnosis and treatment of the patients with COVID-19.”). Participants were asked to rate the items on a 5-point Likert scale (1 = strongly disagree, 5 = strongly agree), with higher scores indicating higher levels of perceived organizational support. The Cronbach’s α of this scale was 0.96 in the present study.

#### PHQ-9

Depression symptoms of healthcare workers in the present study were measured using the well-established screening tool PHQ-9 [45]. Participants were asked to rate items on a 4-point scale (0 = not at all, 3 = nearly every day). The total score is the sum of all items and ranges from 0 to 27. The cut-off point of the Chinese version of the PHQ-9 is 7, with scores over 7 indicating depression [46]. The Chinese version of the PHQ-9 has good reliability and validity and has been used in Chinese samples in previous research [46]. The Cronbach’s α of this scale was 0.91 in the present study.

#### Gad-7

This self-report scale for anxiety symptoms [47] includes 7 items on a 4-point Likert scale ranging from 0 (not at all) to 3 (nearly every day). This study uses the Chinese version of the GAD-7. The cut-off point of the Chinese version of the GAD-7 is 10, with scores over 10 indicating generalized anxiety disorder [48, 49]. The reliability and validity of the Chinese version have been examined in previous research [50]. The Cronbach’s α of this scale was 0.92 in the present study.

#### MBI-GS

The 15-item MBI-GS was used to assess the burnout of healthcare workers under the impact of COVID-19 infection prevention and control. This version is adapted from the original MBI and is considered suitable for occupational groups other than human services and education [51]. There are three dimensions in total, including five questions in the dimension of exhaustion (e.g., “I feel used up at the end of the workday.”), four in cynicism (e.g., “I doubt if my work is meaningful.”), and six in professional efficacy (e.g., “I feel confident that I’m effective at getting things done.”) [52]. Participants were asked to rate items on a 7-point scale (0 = never, 6 = every day). The degree of burnout is determined to be high when the scores in exhaustion and cynicism are high while the score in professional efficacy is low. The total score of the scale was used in data analyses. The Chinese version of the MBI-GS has high reliability and validity among Chinese medical professionals [53–55]. The Cronbach’s αs of the total scale and the three subscales ranged from 0.90–0.92 in the present study.

### Procedures

The present study has been approved by the research ethics committee of Beijing Anding Hospital. Healthcare workers were recruited from 22 tertiary hospitals in Beijing through posters in October 2020. Stratified sampling was used to ensure that healthcare workers working in the frontline and second line during the outbreak period were both included. Participants were asked to scan a QR code and to complete the questionnaires online. A total of 3477 healthcare workers filled out questionnaires and provided effective data for analyses.

### Data analysis

SPSS 18.0 was used to analyse data. Descriptive statistics were used to understand the demographic characteristics and the concentration trends of the main variables. A series of ANOVAs were used to compare group differences in epidemic-related job stressors and psychological wellbeing. Hierarchical multiple regressions were run to examine the predictive effects of epidemic-related job stressors, social support, and organizational support on psychological symptoms and the moderating effects of social support and organizational support.

## Results

### Characteristics of the study sample and epidemic-related job stressors

A series of one-way ANOVAs were conducted to examine the demographic differences in perceived job stressors under the impact of COVID-19 epidemic prevention and control measures. The results yielded a significant difference across professions, *F* (3, 3473) = 13.12, *p* < 0.001. Specifically, doctors (*M* = 37.09, *SD* = 9.19) and medical technicians (*M* = 37.05, *SD* = 9.91) reported the highest level of epidemic-related job stressors, followed by administration staff (*M* = 35.87, *SD* = 10.18) and nurses (*M* = 34.84, *SD* = 10.17). There was also a significant gender difference, *F* (1, 3475) =6.70, *p* = 0.011. Males (*M* = 36.65, *SD* = 9.82) reported significantly higher levels of epidemic-related stressors than females (*M* = 35.60, *SD* = 9.98). In addition, the age difference was significant, *F* (3, 3473) =35.66, *p* < 0.001. Healthcare workers in the age groups of 40–50 and above 50 reported the highest levels of epidemic-related job stressors (*M* = 38.11, *SD* = 9.66 and *M* = 39.05, *SD* = 9.95, respectively), followed by participants in the age group of 30–40 (*M* = 35.28, *SD* = 9.99) and those in the age group of 20–30 (*M* = 33.90, *SD* = 9.66), *p* < 0.01. Participants with varying lengths of working experience were not significantly different in the perceived level of epidemic-related job stressors.

Significant differences in epidemic-related job stressors were also found in different exposure risk groups (*F* (3, 3473) = 20.15, *p* < 0.001). The results of post hoc tests revealed that participants in the lower exposure risk group reported significantly higher stressor scores (*M* = 36.81, *SD* = 9.91) than medical team members in the small epidemic in Beijing (*M* = 33.50, *SD* = 9.46) and medical team members in the Hubei outbreak (*M* = 33.94, *SD* = 8.25), *p* < 0.01. Healthcare workers in the higher exposure risk group also reported higher stressor scores (*M* = 35.70, *SD* = 10.24) than medical team members in the small epidemic in Beijing (*M* = 33.50, *SD* = 9.46).

### Psychological symptoms of healthcare workers

The means of psychological symptoms of healthcare workers are reported in Table 2. Using 10 as the cut-off point of the GAD-7 and 7 as the cut-off point of the PHQ-9, 145 participants (4.2%) were identified as having significant anxiety symptoms, and 797 (22.9%) participants were identified as experiencing significant depression symptoms.

**Table 2:** Psychological symptoms of healthcare workers

	Lower exposure risk group (*n* = 1890)	Higher exposure risk group (*n* = 820)	Medical team members in a small epidemic in Beijing (*n* = 700)	Medical team members in Hubei outbreak (*n* = 67)	Statistics
Anxiety					
M	3.26	3.42	2.62	3.58	*F* (3, 3473) =8.65, *p* < 0.001
SD	3.45	3.49	2.95	3.15	
Depression					
M	3.86	4.02	3.14	4.22	*F* (3, 3473) =7.14, *p* < 0.001
SD	4.25	4.20	3.54	4.12	
Burnout					
M	31.15	31.55	28.55	28.85	*F* (3, 3473) =7.37, *p* < 0.001
S D	14.62	13.69	13.56	13.61	

One-way ANOVAs were conducted to test the demographic differences of symptoms. There were significant differences in anxiety scores among the professional groups, *F* (3, 3473) = 3.66, *p* < 0.05. Doctors reported greater anxiety (*M* = 3.50, *SD* = 3.45) than other professions (for nurses, *M* = 3.08, *SD* = 3.36; for technicians, *M* = 3.14, *SD* = 3.28; for administrative staff, *M* = 2.89, *SD* = 3.36). Age difference in anxiety was significant, *F* (3, 3473) = 14.43, *p* < 0.001, healthcare workers under 30 years old reported lower levels of anxiety (*M* = 2.48, *SD* = 2.90) compared with other age groups (for the group of 31–40, *M* = 3.25, *SD* = 3.32; for the group of 41–50, *M* = 3.56, *SD* = 3.60; for the group ≥51, *M* = 3.48, *SD* = 3.91). The effect of working experience on anxiety was also significant, *F* (3, 3473) =8.47, *p* < 0.001. Healthcare workers with less than 5 years of working experience had significantly lower anxiety scores (*M* = 2.61, *SD* = 3.19) than those who had been practicing for 11–20 years and 21–30 years (for the group working for 11–20 years, *M* = 3.34, *SD* = 3.40; for the group working for 21–30 years, *M* = 3.65, *SD* = 3.69). Gender and income had no significant effect on the anxiety scores reported.

In terms of demographic differences in depression, significant differences were found across age groups (*F* (3, 3473) =15.72, *p* < 0.001) and groups with various lengths of working experience (*F* (3, 3473) =8.62, *p* < 0.001). Healthcare workers under 30 years old reported lower depression scores than other age groups (for the group below 30 years old, *M* = 2.98, *SD* = 3.54; for the group of 31–40, *M* = 3.76, *SD* = 4.04; for the group of 41–50, *M* = 4.43, *SD* = 4.40; for the group above 51, *M* = 3.90, *SD* = 4.76). Healthcare workers who had been practicing for less than 5 years had significantly lower depression scores than those who had been practicing for 10 to 30 years (for the group working less than 5 years, *M* = 3.11, *SD* = 3.99; for the group working for 11–20 years, *M* = 3.90, *SD* = 4.04; for the group working for 21–30 years, *M* = 4.41, *SD* = 4.41).

Healthcare workers differing in age groups and professions reported significant differences in levels of burnout (*F* (3, 3473) =3.91, *p* = 0.008 and *F* (3, 3473) =5.58, *p* = 0.001, respectively). Healthcare workers in the 31–40 and 41–50 age groups reported higher burnout scores than other age groups (for the group below 30 years old, *M* = 29.59, *SD* = 14.02; for the 31–40 age group, *M* = 31.21, *SD* = 14.31; for the 41–50 age group, *M* = 31.30, *SD* = 13.91; and for the group above 51 years old, *M* = 29.13 *SD* = 15.00). In addition, administrative staff reported a lower level of burnout (*M* = 27.62, *SD* = 14.96) than the other three professions (for doctors, *M* = 31.66, *SD* = 13.40; for nurses, *M* = 30.70, *SD* = 14.21; for medical technicians, *M* = 30.65, *SD* = 14.80).

Group differences in symptoms for healthcare workers with different exposure risks were also examined. The group difference in anxiety was significant, *F* (3, 3473) =8.65, *p* < 0.001. As displayed in Table 2, members of medical teams in the small epidemic in Beijing reported lower levels of anxiety compared with healthcare workers in the lower exposure risk group and those in the higher exposure risk group.

The group differences in depression and burnout were also significant and showed similar patterns (for depression, *F* (3, 3473) =7.14, *p* < 0.001; for burnout, *F* (3, 3473) =7.37, *p* < 0.001). Members of medical teams in the small epidemic in Beijing reported lower depression scores and burnout compared with healthcare workers in the lower exposure risk group and those in the higher exposure risk group. The means and SDs of depression and burnout of each group are shown in Table 2.

### Correlations among Main variables

The results of correlations among stressors, organizational support, social support, depression, anxiety, and burnout are displayed in Table 3. The overall epidemic-related job stressor scores were negatively correlated with perceived organizational support (*r* = − 0.40, *p* < 0.01) and social support (*r* = − 0.39, *p* < 0.01) and positively correlated with depression (*r* = 0.38, *p* < 0.01), anxiety (*r* = 0.36, *p* < 0.01) and burnout (*r* = 0.54, *p* < 0.01). Both organizational support and social support had significant negative correlations with depression, anxiety, and burnout.

**Table 3:** correlations among main variables

	1	2	3	4	5	6	7	8	9
1.total score of epidemic-related job stressors	1								
2.stressors related to infection prevention and control measures	0.94^**^	1							
3. high work load	0.68^**^	0.47^**^	1						
4.infection anxiety	0.64^**^	0.47^**^	0.31^**^	1					
5.Organization support	−.40^**^	−0.44^**^	−0.17^**^	−0.19^**^	1				
6.Social support	−.39^**^	− 0.43^**^	− 0.12^**^	− 0.21^**^	.54^**^	1			
7.Depression	.38^**^	0.38^**^	0.22^**^	0.21^**^	−.27^**^	−.33^**^	1		
8.Anxiety	.36^**^	0.36^**^	0.20^**^	0.23^**^	−.24^**^	−.31^**^	.84^**^	1	
9.Burnout	.54^**^	0.57^**^	0.26^**^	0.27^**^	−.52^**^	−.59^**^	.48^**^	.45^**^	1

### Modeling testing

Hierarchical multiple regressions were run to examine the hypothesized models. Anxiety, depression, and burnout were used as the dependent variable separately. Demographic variables including gender, age, length of working experience, and professions were put in the first block of the regression as covariates; job exposure risk (lower risk vs. higher risk) was put in the second block; the frontline working experience in the outbreak stage of COVID-19 was put in the third block; epidemic-related job stressor was put next; social support and organizational support were included in the fifth block, and the interaction terms of job stressor and organizational support and job stressor and social support were put in the final block. All the variables had been standardized. The results of hierarchical regressions are illustrated in Table 4.

**Table 4:** Results of hierarchical multiple regressions

Depended variable	Independent variable	step 1		step 2			step 3			step 4			step 5			step 6		
		R^2^	β	R^2^	ΔR^2^	β	R^2^	ΔR^2^	β	R^2^	ΔR^2^	β	R^2^	ΔR^2^	β	R^2^	ΔR^2^	β
Anxiety	Age	.01^**^	.14^*^	.012	.001	.143^*^	.016	.004^**^	.148^**^	.134	.118^**^	.114^*^	.170	.036^**^	.117^*^	.174	.004^**^	.114^*^
	Work year		−.04^*^			−.048			−.060			−.080			−.083			−.077
	Gender		.04^*^			.035^*^			.030			.038^*^			.046^**^			.044^**^
	Career 1		−.05^*^			−.051^*^			−.052^**^			−.032			−.033			−.035
	Career 2		−.02			−.029			−.027			−.026			−.034			−.038
	Career 3		−.03			−.031			−.010			.009			.018			.013
	JER					.031			.021			.011			.011			.012
	FEW 1								−.069^**^			−.040^*^			−.025			−.026
	FWE 2								.011			.023			.028			.029
	Stressor											.352^**^			.267^**^			.274^**^
	OS														−.039^*^			−.037
	SS														−.187^**^			−.185^**^
	SS × Stressor																	−.049^**^
	OS×Stressor																	−.025
Depression	Age	.010^**^	.112^*^	.011	.001^*^	.116^*^	.014	.003^**^	.120^*^	.142	.128^**^	.085	.186	.045^**^	.091	.193	.007^**^	.086
	Work year		−.014			−.019			−.029			−.050			−.054			−.047
	Gender		.036^*^			.035^*^			.031			.039^*^			.048^**^			.046^**^
	Career 1		−.039			−.039^*^			−.040^*^			−.020			−.020			−.022
	Career 2		.003			−.002			−.001			.001			−.009			−.014
	Career 3		−.016			−.014			.004			.024			.036			.029
	JER					.039^*^			.031			.021			.021			.022
	FWE 1								−.059^**^			−.029			−.011			−.013
	FWE 2								.012			.024			.030			.032^*^
	Stressor											.366^**^			.268^**^			.276^**^
	OS														−.062^**^			−.060^**^
	SS														−.197^**^			−.194^**^
	SS × Stressor																	−.056^**^
	OS×Stressor																	−.042^*^
Burnout	Age	.005^**^	−.030	.005	.000	−.030	.012	.007^**^	−.020	.310	.298^**^	−.075	.511	.201^**^	−.044	.514	.003	−.040
	Work year		.044			.043			.027			.000			−.023			−.028
	Gender		.033			.032			.023			.035^*^			.050^**^			.052^**^
	Career 1		−.088^**^			−.088^**^			−.091^**^			−.058^**^			−.051^**^			−.049^**^
	Career 2		−.035			−.036			−.036			−.035			−.052^**^			−.048^**^
	Career 3		−.048			−.048			−.018			.014			.044^*^			.047^*^
	JER					−.007			−.006			−.02^**^			−.022^**^			−.022
	FWE 1								−.085^**^			.038^*^			.002			.004
	FWE 2								−.035^*^			.014			.004			.003
	Stressor											.56^**^			.337^**^			.329^**^
	OS														−.365^**^			−.368^**^
	SS														−.209^**^			−.213^**^
	SS × Stressor																	.041^**^
	OS×Stressor																	.018

Note. Career 1- Career 3 were dummy variables of professions; FWE 1 and FWE 2 were dummy variables of FWE (frontline working experience), FWE1 = whether medical rescue team members in Beijing epidemics, FWE2 = whether assigned to Hubei during the outbreak period

*JER* Job exposure risk, *OS* Organization Support, *SS* Social Support

^*^p < 0.05

^**^p < 0.01

For the regression using anxiety as the dependent variable, the whole model explained 17.5% of the variance in anxiety. The predictive effect of epidemic-related job stressors was significant after controlling demographic variables, job exposure risk and frontline working experience in the outbreak stage, *F* (1, 3418) = 469.03, *ΔR*^*2*^ = 0.12, *p* < 0.001. The effects of perceived organizational support and social support were also significant over the effect of job stressors, *F* (2, 3416) = 74.26, *ΔR*^*2*^ = 0.04, *p* < 0.001. The predictive effect of interaction terms was significant, *F* (2, 3414) = 8.678, *ΔR*^*2*^ = 0.004, *p* < 0.001. In the final regression model, the significant predictors were age (*β* = 0.11, *p* < 0.01), gender (*β* = 0.04, *p* < 0.01), epidemic-related job stressors (*β* = 0.27, *p* < 0.01), social support (*β* = − 0.19, *p* < 0.01), and the interaction term of stressors and social support (*β* = − 0.05, *p* < 0.01).

For the regression using depression as the dependent variable, the model explained 19.3% of the variance. The effect of job stressors was significant after controlling demographic variables, job exposure risk and frontline working experience in the outbreak stage, *F* (1, 3418) = 510.77, *ΔR*^*2*^ = 0.13, *p* < 0.001. The predictive effects of perceived organizational support and social support were also significant over the effect of job stressors, *F* (2, 3416) = 93.98, *ΔR*^*2*^ = 0.05, *p* < 0.001. The predictive effect of interaction terms was significant, *F* (2, 3414) = 15.51, *ΔR*^*2*^ = 0.007, *p* < 0.001. In the final regression, the significant predictors of depression included gender (*β* = 0.05, *p* < 0.01), frontline working experience in the Hubei outbreak (β = 0.03, *p* < 0.05), job stressors (*β* = 0.28, *p* < 0.01), perceived organizational support (*β* = − 0.06, *p* < 0.01), social support (*β* = − 0.19, *p* < 0.01), the interaction term of job stressors and social support (*β* = − 0.06, *p* < 0.01) and the interaction term of job stressors and organizational support (*β* = − 0.04, *p* < 0.05).

For the regression using burnout as the dependent variable, the final model explained 51.4% of the variance in burnout. The effect of job stressors was significant after controlling demographic variables, job exposure risk, and frontline working experience in the outbreak stage, *F* (1, 3329) =1443.26, *ΔR*^*2*^ = 0.298, *p* < 0.001. The predictive effects of perceived organizational support and social support were also significant over the effect of job stressors, *F* (2, 3327) =685.73, *ΔR*^*2*^ = 0.20, *p* < 0.001. The predictive effect of interaction terms was significant, *F* (2, 3325) = 9.34, *ΔR*^*2*^ = 0.003, *p* < 0.001. In the final regression, the significant predictors of burnout included gender (*β* = 0.05, *p* < 0.01), the three dummy codes of professions (*βs* were − 0.05, − 0.05, 0.05, respectively, *ps* < 0.05), job stressors (*β* = 0.33, *p* < 0.01), perceived organizational support (*β* = − 0.37, *p* < 0.01), social support (*β* = − 0.21, *p* < 0.01), and the interaction term of job stressors and social support (*β* = 0.04, *p* < 0.01).

### Simple slope testing for significant interaction effects

As indicated by the results of hierarchical multiple regressions, the moderating effect of social support was significant in the relationships of job stressors-anxiety, job stressors-depression, and job stressors-burnout; the moderating effect of organizational support was significant in the relationships of job stressors-depression. Therefore, simple slope analyses were conducted to further examine the significant moderating effects.

**Fig. 2:**
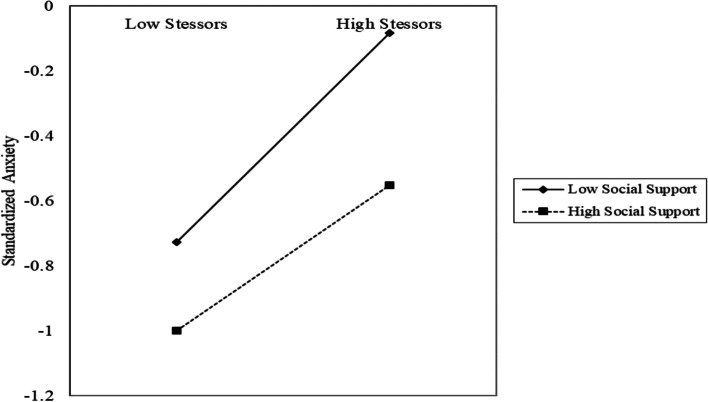
The relationship between stressors and anxiety at high and low levels of social support

**Fig. 3:**
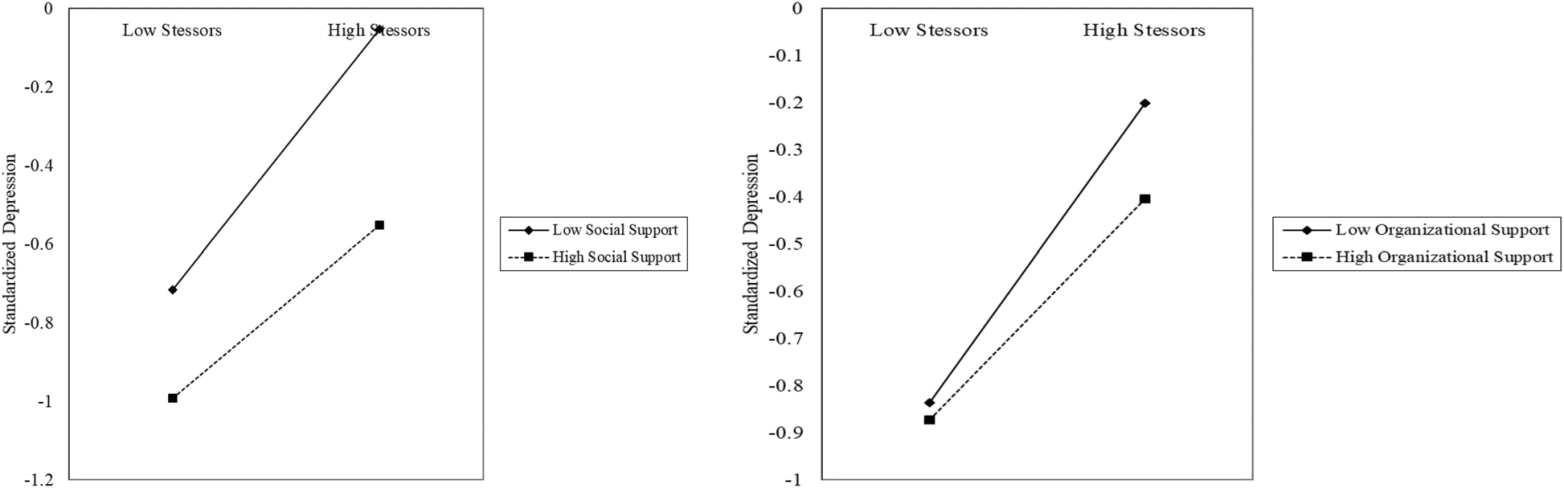
The relationship between stressors and depression at high and low levels of social and organizational support

**Fig. 4:**
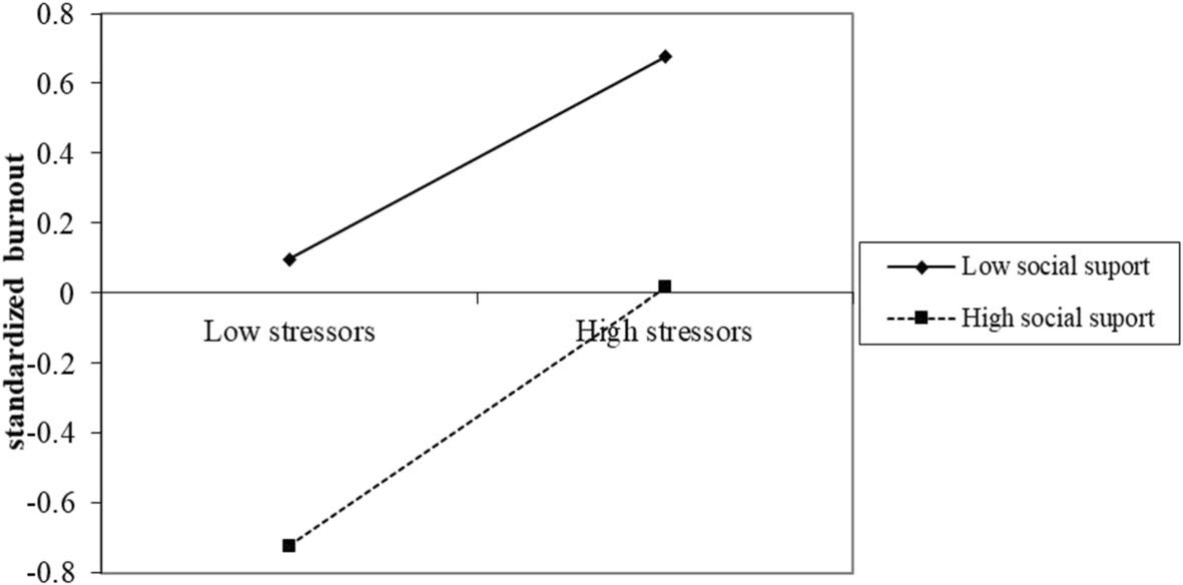
The relationship between stressors and burnout at high and low levels of social support

As shown in Fig. 2, the predictive effect of epidemic-related job stressors on anxiety was more pronounced among healthcare workers with a lower level of social support. Similarly, the predictive effect of epidemic-related job stressors on depression was greater among healthcare workers with a lower level of social support and organizational support (seen in Fig. 3). However, as shown in Fig. 4, the predictive effect of epidemic-related job stressors on burnout was less pronounced among healthcare workers with a higher level of social support.

## Discussion

The present study aimed to investigate job stressors, well-being, and burnout of Chinese healthcare workers under regular epidemic prevention and control measures and to examine the effects of epidemic-related stressors, perceived social support, and organizational support on well-being and burnout in this population. Healthcare workers working in high exposure risk departments such as COVID-19 wards and infectious disease departments and those working in other low exposure risk departments were involved. The effects of previous frontline working experience on current psychological well-being were also examined.

The results revealed that medical team members of the Beijing epidemic (in June 2020) reported the lowest levels of burnout, depression, and anxiety. Medical team members in the Hubei outbreak (Jan 2020 to April 2020) did not exhibit significantly higher burnout or psychological symptoms. However, the well-being of healthcare workers who did not participate in medical rescue during the pandemic was reported to be relatively poorer. This result is consistent with some research comparing the mental health of frontline and second-line healthcare workers [56, 57] but opposite to the results of others [39, 58, 59]. It might be that the frontline healthcare workers had the opportunities to learn more about the virus and disease, built greater efficacy when working with top teams, received greater respect from the whole society, and experienced the greater value of the job. In contrast, second-line healthcare workers reported a lower level of organizational support, which might be a reason for experiencing more psychological symptoms, including anxiety and depression. Thus, it seems that frontline working experience is not necessarily related to negative long-term effects on mental health.

Several reasons could explain the low level of well-being of healthcare workers with lower job exposure risk. First, these healthcare workers were not familiar with infection prevention and control measures in their previous work; therefore, they needed to make the greatest effort to adapt to the new situation. Second, they face the greatest uncertainty in daily work. The proportion of patients with COVID-19 in their departments should be smaller than those in departments such as respiratory medicine, infectious disease, and fever clinics. However, they need to be well prepared for minor probability events. As inspired by the results, burnout and well-being of healthcare workers with low-er-exposure risk jobs should not be overlooked by organizations.

In line with the rationale of the JD-R model, epidemic-related job stressors, novel job demands in the period of the COVID-19 pandemic, were positively associated with anxiety, depression, and burnout. Therefore, Hypothesis 1 was supported. In addition to work overload and infection anxiety, which were important job stressors in the outbreak period of COVID-19, stressors related to regular infection prevention and control measures such as discomfort caused by personal protection equipment, perceived competence, and interpersonal stressors also contributed to burnout, anxiety, and depression. This is consistent with previous findings that hospital infection prevention and control measures predicted burnout [60, 61]. Thus, it is crucial to notice these potential adverse effects and take measures to help healthcare workers cope with this novel job stressor.

Consistent with hypothesis 2, protective effects of perceived social support and organizational support on the well-being of healthcare workers were found. This is in line with earlier research in which researchers found that social and organizational support moderated the relationship between work-related stress and negative emotions [62], and both of those supports contributed to better well-being, even during the COVID-19 pandemic [63]. Although both support systems were considered important job resources, previous research seldom examined their effects together. Our results indicated that both social support and organizational support had independent predictive effects on the well-being of healthcare workers, with social support seeming more closely related to depression and anxiety and organizational support being more closely related to burnout.

In addition to the direct protective effect on well-being, social support buffered the adverse effect of epidemic-related job stressors on anxiety and depression, and organizational support buffered the effects of job stressors on depression. These results were in line with the JD-R model and supported hypothesis 3. However, the role of social support in the relationship between epidemic-related job stressors and burnout was contrary to the JD-R model. That is, the effect of job stressors on burnout was enhanced in healthcare workers with higher social support. The reversed buffering effect has also been reported in previous research [64, 65]. Chisholm et al. (1986) proposed that the buffering effect of social support is highly selective [64]. Specifically, support from people at the worksite is the most important to relieve the effects of job stressors. It might be that social support, which was measured as the individual support system, is not directly associated with the content of one’s work and has relatively limited effectiveness on coping with job stressors. When job stress is low, social support might be more effective, whereas it is not as effective in circumstances of high job stress.

The present study has practical implications for promoting the occupational health of healthcare workers in the post-pandemic period of COVID-19. First, healthcare workers with a higher risk of psychological problems in this period were identified. Doctors, females, people aged 30 to 50, and those who did not participate in medical rescue during the outbreak period and working in departments with lower exposure risk reported higher levels of burnout, anxiety, and depression. Therefore, their mental health needs should be more attended to. Second, in addition to work overload and infection anxiety, stressors related to epidemic prevention and control had a significant effect on burnout and well-being. Therefore, reducing this job stressor by simplifying the work routine or assigning work responsibilities might be helpful. Third, in line with opinions of Chirico and Ferrari (2021) [66], we argue that protective measures should be implemented at the individual and organizational levels. The individual social system is beneficial for their well-being and reducing burnout. Therefore, healthcare workers should be encouraged to identify their social support system and seek support from their families and friends under high work stress. It is also beneficial for individuals with religions to participate in spiritual activities [67]. In the period of regular epidemic prevention and control, support from the organization is particularly important. Infection anxiety could be reduced if organizations provide adequate personal protective equipment and informative training about how to use them properly [58]. It is also helpful to provide routine health surveillance for healthcare workers [68]. Interpersonal stressors related to infection and control measures could be reduced in a supportive organizational climate [69, 70].

The findings of this study need to be considered within several limitations. First, given that infection prevention and control measures could be changed according to the changing epidemic trend, a cross-sectional investigation could not reflect the longitudinal change in job stressors and well-being. It would be necessary to conduct a longitudinal study to explore the trajectories of job stressors and the well-being of healthcare workers at different times with varying severities of the epidemic. Second, this study measured the subjective perception of organizational support; however, it did not investigate how objective supportive measures taken by organizations could influence burnout and the well-being of healthcare workers. Including objective supportive measures in future studies could be helpful to clarify the effects of objective support and perceived support and would have greater practical implications for the provision of supportive measures.

## Conclusions

The results of the present study confirmed the adverse effect of epidemic-related job stressors on predicting burnout, depression, and anxiety symptoms in healthcare workers during the post-pandemic period under regular epidemic prevention and control measures. It has illustrated the important protective effects of social support and organizational support to buffer the negative effect of work-related stressors. Managers should be more aware of psychological conditions among healthcare workers under the impact of COVID-19 infection prevention and control measures, take steps to reduce epidemic-related stressors, and provide external support to reduce anxiety, depression, and burnout among healthcare workers.

## Data Availability

The datasets used and analyzed during the current study are available from the corresponding author on reasonable request. The data is not available publicly because the authors want to know who are interested in this study and why during the private communication.
